# Lymph Node Dissection in Curative Gastrectomy for Advanced Gastric Cancer

**DOI:** 10.1155/2011/748745

**Published:** 2011-06-14

**Authors:** Shigeyuki Tamura, Atsushi Takeno, Hirofumi Miki

**Affiliations:** Department of Surgery, Kansai Rosai Hospital, 1-69 3-Chome, Inabasou, Amagasaki, Hyogo 660-8511, Japan

## Abstract

Gastric cancer is one of the most common causes of cancer-related death worldwide. Surgical resection with lymph node dissection is the only potentially curative therapy for gastric cancer. However, the appropriate extent of lymph node dissection accompanied by gastrectomy for cancer remains controversial. In East Asian countries, especially in Japan and Korea, D2 lymph node dissection has been regularly performed as a standard procedure. In Western countries, surgeons perform gastrectomy with D1 dissection only because D2 is associated with high mortality and morbidity compared to those associated with D1 alone but does not improve the 5-year survival rate. However, more recent studies have demonstrated that western surgeons can be trained to perform D2 lymphadenectomies on western patients with a lower morbidity and mortality. When extensive D2 lymph node dissection is preformed safely, there may be some benefit to D2 dissection even in western countries. In this paper, we present an update on the current literature regarding the extent of lymphadenectomy for advanced gastric cancer.

## 1. Introduction

 Gastric cancer is one of the most common causes of death worldwide [1]. Although the prognosis of patients with advanced gastric cancer has improved with the introduction of effective chemotherapy [2] or adjuvant radiotherapy [3], surgical resection remains the primary therapeutic modality for curable advanced cancer. With regard to surgical procedure, dissection of regional LN is regarded an important part of en bloc resection for gastric cancer. However, there are significant differences in the extent of lymphadenectomy preformed by surgeons in different countries. 

In Japan, D2 dissection has been recommended as standard practice since the 1960s [4]. East Asian surgeons, especially Japanese and Korean surgeons, routinely performed gastrectomy with D2 dissection. However, most Western surgeons perform gastrectomy with only D1 dissection, because D1 was associated with less mortality and morbidity than D2 in prospective randomized trials preformed in the Netherland and the UK concluded that there was no survival benefit for D2 over D1 lymph node dissection [5, 6]. However, there were significant problems with these studies, including a high morbidity and mortality rate in the D2 group associated with inadequate surgical training, with inadequate dissection of D2 and with the frequent performance of distal pancreatectomy and splenectomy in the D2 group, which is now considered unnecessary [7].

 More recent studies have demonstrated that western surgeons at experienced centers can be trained to perform D2 gastrectomy for selected western patients with low morbidity and mortality [8–10]. There may be some benefits to D2 gastrectomy when performed safely, but this assertion requires further validation to establish the global standard in gastrectomy.

 In this paper, we describe an update on the current literature regarding the extent of lymphadenectomy for advanced gastric cancer.

## 2. Grouping of Lymph Nodes

**Figure 1:**
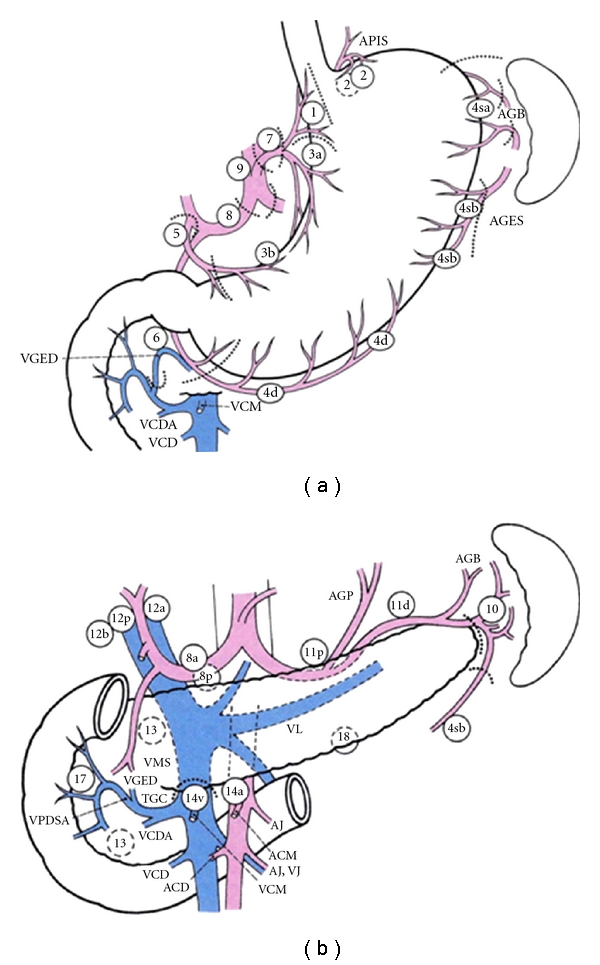
Lymph node station numbers according to the Japanese classification of gastric cancer of the 14th edition reproduced form [12] with permission.

The lymph nodes of the stomach have been arranged into a very useful classification by the Japanese Gastric Cancer Association (JGCA) [11, 12] (Table 1, Figure 1).

According to this classification, lymph nodes surrounding stomach are divided into 20 stations and these are classified into three groups depending upon the location of the primary tumor. This grouping system is based on the results of studies of lymphatic flow at various tumor sites, together with the observed survival associated with metastasis to each nodal station [13]. In this grouping system, the most perigastric LNs (stations nos. 1–6) are defined as group 1, whereas the nodes along the left gastric artery (station no. 7), common hepatic artery (station no. 8), celiac axis (station no. 9), splenic artery (station no. 11) and proper hepatic artery (station no. 12) are defined as group 2. Minor modifications of this grouping system are necessary according to the location of the primary tumor. D1 gastrectomy is defined as dissection of all the Group 1 nodes, and D2 is defined as dissection of all the Group 1 and Group 2 nodes. 

Recently, new Japanese Classification of Gastric Carcinoma [12] and guideline for Diagnosis and Treatment of Carcinoma of the Stomach [14] edited by the Japanese Gastric Cancer Society were published in May and October, 2010 to match to the standard of TNM classification of UICC [15, 16] (Tables 2 and 3).

In this classification, the extent of LN metastasis is divided into three groups according to the number of metastatic LN, not to the *N*-number of the extent of LN metastasis. 

Moreover in this guideline, the main modification about lymph node dissection is that selection of D1 or D2 dissection is prescribed by the kind of gastrectomy, for example, total gastrectomy or distal gastrectomy, not by the location of the primary tumor. It is provided that D1 gastrectomy includes the dissection of the nodes along the left gastric (station no. 7) as well as the perigastric lymph nodes (stations nos. 1–6), regardless of the location of tumor. LNs along the superior mesenteric vein (station no. 14v) are eliminated from D2 dissection for tumor in the lower third of the stomach. 

**Figure 2:**
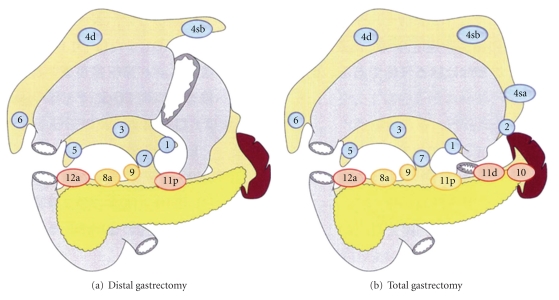
Lymph node dissection according to the Japanese gastric cancer treatment guideline 2010 of the 3rd edition reproduced form [14] with permission. D1 distal gastrectomy consists of LN dissection of station nos. 1, 3, 4sb, 4d, 5, 6, and 7 and D1 total gastrectomy consists of station nos. 1–6 and 7 (blue circle). Yellow circles indicate the lymph nodes that belong to D1+, and red circles indicate those to D2.

In other words, D1 distal gastrectomy consists of LN dissection of station nos. 1, 3, 4sb, 4d, 5, 6, and 7 and D1 total gastrectomy includes station nos. 1–6 and 7 (Figure 2). 

In Japan, although the surgical procedure is performed according to the new guidelines, standard surgery for cN1 or T2 and more cases is defined as gastrectomy with D2 dissection. 

## 3. D1 versus D2

 In Japan, D2 dissection was introduced in the 1960's and gastrectomy with D2 dissection has been regarded as a safe surgical procedure and performed regularly in ordinary general hospitals [4]. Therefore, in Japan, a clinical trial comparing D1 versus D2 would be considered unethical today. 

 However, whether D2 LN dissection in radical gastrectomy should be routinely performed is still unclear in the world. 

 Based on the results of several RCTs comparing D1 and D2/D3 dissection performed in western countries, D2 dissection is not recommended because D2 is associated with high morbidity and mortality rate. 

Two large-scale RCTs wereperformedby the Dutch Gastric Cancer Group [5, 17–19] and Medical Research Council Gastric Cancer Surgical Group [6, 20] (Table 4). The RCT by the Dutch group was performed between 1989 and 1993 and involved 711 patients from 80 hospitals but excluded 285 patients who had received palliative treatment [5]. The RCT by the British group was performed between 1987 and 1994 and involved 400 patients but excluded 337 patients based on staging laparoscopy demonstrating advanced disease [6]. 

 The stage distribution in the Dutch RCT was slightly less advanced than that in the British study; UICC stage I tumors comprised 43% and 35% of the total, respectively, and T3 tumors comprised 44 and 27%. 

In the Dutch trial, D2 patients demonstrated higher postoperative morbidity (43% versus 25% for D1: *P* < .001) and higher morbidity (10% versus 4% for D1: *P* < .004). Overall 5-year survival rates were similar in the D1 and D2 groups (45% for D1 and 47% for D2).

The hazard ratio (HR) comparing the risk of death within 5 years after D2 surgery to that of 5 years after D1 surgery was 1.00 (95% confidence interval (95% CI), 0.82–1.22) [5]. However, at 11 years, survival rates were 30% for D1 and 35% for D2 (*P* = .53). When hospital deaths were excluded, survival rates were 32% for D1 (*n* = 365) and 39% for D2 (*n* = 299) and the relative risks of these patients favored the D2 surgery group (*P* = .07) [17].

Low-quality surgery due to a very low hospital volume could explain why D2 surgery was not beneficial, along with high hospital mortality in that series. About 50% of the patients in the D2 group did not undergo lymph node dissection at all stations that should have been resected. However, 6% of the patients in the D1 group underwent dissection of more stations that would not been resected in D1 surgery. These factors could have led to the limited difference in outcomes, between D1 and D2 surgery [18].

Recently, 15-year follow-up results of a randomized nationwide Dutch D1D2 trial were reported. The overall 15-year survival was 21% (82 patients) for the D1 group and 29% (92 patients) for the D2 group (*P* = .34). The gastric-cancer related death rate was significantly higher in the D1 group (48%, 182 patients) compared with that in the D2 group (37%, 123 patients), whereas death due to other diseases was similar in both groups [19]. 

The authors indicated in the interpretation that because a safer, spleen-preserving D2 resection technique had become available in high-volume centers, D2 lymphadenectomy should be the recommended surgical approach for patients with resectable (curable) gastric cancer. 

In the British study, postoperative complications were significantly higher in the D2 group (46%) than in the D1 group (28%; *P* < .001), and the postoperative mortality was also significantly higher in the D2 group (13%) than in the D1 group (6.5%; *P* = .04) [6].

 In this study, splenectomy was performed for many patients with distal gastrectomy and pancreaticosplenectomy was carried out in 56% of patients allocated to the D2 group and 4% of the D1 group. The high frequency of postoperative complications was influenced by the excessive surgery, which contributed to a misunderstanding of the definition of D2 gastrectomy defined by the Japanese Gastric Cancer Association. The 5-year survival rate was 33% in the D1 group and 35% in the D2 group, which did not significantly differ between the two groups [20]. 

 Unlike these two large European trials, the Italian Gastric Cancer Study Group (IGCSG) has shown the safety of D2 dissection with pancreas preservation in a one-arm phase I-II trial [9]. Between 1994 and 1996, 191 eligible patients were entered in the study. The overall morbidity rate was 20.9%. Surgical complications were observed in 16.7% of patients and reoperation was necessary in six patients and was successful in all cases. The overall hospital mortality rate was 3.1%; it was higher after total gastrectomy (7.46%) than after distal gastrectomy (0.8%). This study concluded that postoperative morbidity and mortality rates were favorably comparable to those reported after the standard Western gastrectomy and that the more extensive Japanese procedure with pancreas preservation can be regarded as a safe radical treatment for gastric cancer in selected Western patients treated at experienced centers. 

A small-scale RCT comparing of the morbidity and mortality of D1 to D2 gastrectomy was performed by IGCSG [10].

 Of 162 patients randomized, 76 were allocated to D1 and 86 to D2 gastrectomy. The overall postoperative morbidity rate was 13.6%. Complications developed in 10.5% of patients after D1 and in 16.3% of patients after D2 gastrectomy. This difference was not statistically significant (*P* < .29). The overall postoperative mortality rate was 0.6% (one death); it was 1.3% after D1 and 0% after D2 gastrectomy. This study confirmed that, at very experienced centers, morbidity and mortality after extended gastrectomy could be as low as those after D1 gastrectomy.

 Another single-institutional small-scale RCT has reported from Taiwan that there were no significant differences in the postoperative and mortality between patients undergoing D3 and D1 gastrectomy [21, 22]. This was the only trial that showed a significantly higher 5-year disease-specific survival in patients with D3 surgery than in those with D1 surgery (Table 4). 

Therefore, D2 gastrectomy is becoming accepted as a safe treatment for gastric cancer at experienced centers, in western countries.

## 4. D2 versus D3

 In Japan, gastrectomy with more radical extended lymphadenectomy had been performed since 1980's at many specialized centers in order to improve the prognosis of patients with advanced gastric cancer [23–26]. The incidence of microscopic metastasis in the paraaortic nodes (section no. 16) in patients with gastrectomy undergoing D3 lymph node dissection ranged from 6% to 33%, and the 5-year survival rate had been reported to range from 12% to 23% in patients undergoing gastrectomy with D3 dissection. Extending these previous findings regarding the favorable results of D3 dissection, the Japanese Clinical Oncology Group (JCOG) conducted a randomized clinical trial between 1995 and 2001 to compare D2 gastrectomy alone with D2 plus paraaortic lymph node dissection (PAND) [27]. A total of 523 patients with T2b, T3, and T4 gastric cancer were registered and randomly assigned to D2 alone group (263 patients) or D2 plus PAND group (260 patients). 

 The rates of surgery-related complications among patients assigned to D2 lymphadenectomy alone and those assigned to D2 lymphadenectomy plus PAND were 20.9% and 28.1%, respectively (*P* = .07). There were no significant differences between the two groups in the frequencies of anastomotic leakage, pancreatic fistula, abdominal abscess, pneumonia, or death from any cause within 30 days after surgery (the mortality was 0.8% in each group). The 5-year overall survival rate was 69.2% for the group assigned to D2 lymphadenectomy alone and 70.3% for the group assigned to D2 lymphadenectomy plus PAND; the hazard ratio for death was 1.03. Moreover, there were no significant differences in recurrence-free survival between the two groups.

 Recently, meta-analyses of D2 lymphadenectomy versus D2 with PAND were reported [28]. Three RCTs including the PGCSG study in Poland [29], EASOG study in Japan, Korea, and Chinese Taiwan area [30, 31], and JCOG-9501 study in Japan [27] were eligible (Table 5). Another analysis included 4 RCTs and 4 nonrandomized studies were identified [32]. These meta-analyses showed that D2+ PAND can be performed as safely as a standard D2 resection without increasing postoperative mortality but failed to benefit overall survival in patients with advanced gastric cancer.

 Gastrectomy with D2 lymphadenectomy plus PAND cannot be recommended as a routine practice for the surgical treatment of gastric cancer.

## 5. Mediastinal Lymph Node Dissection for Gastric Cancer

For patients with esophageal invasion from gastric cancer, it is necessary to perform mediastinal resection included the lower esophagus and the periesophageal lymph nodes and to confirm that the esophageal cut end is negative by performing histological examination using frozen section as necessary [33]. Conventionally, this mediastinal procedure was done through the left thoracoabdominal approach (LTA), because the frequency of lymph node metastasis was reported to be high with about 20–40% and an adequate margin from the tumor could be secured. However, a mediastinal procedure was enabled through the abdominal-transhiatal approach (TH) with advances in surgical methods using a circular stapler in recent years.

 In Japan, an RCT comparing LTA versus TH for Siewert type II and III tumors with esophageal invasion of 3 cm or less was carried out by JCOG [34]. Between 1995 and 2003, 167 patients were enrolled from 27 Japanese hospitals and randomly assigned to TH (*n* = 82) or LTA (*n* = 85), although the projected sample size was 302. After the first interim analysis, the predicted probability of LTA having a significantly better overall survival than TH at the final analysis was only 3.65%; therefore, the trial was closed. The 5-year overall survival was 52.3% in the TH group and 37.9% in the LTA group. The hazard ratio of death for LTA compared with TH was 1.36 (0.89–2.08, *P* = .92). Three patients died in hospital after LTA but none after TH. Morbidity after LTA was worse than that after TH with rates of 49% and 34%, respectively. 

This study concluded that LTA could not be performed for gastric cancer with esophageal invasion of 3 cm or less, because LTA did not improve survival compared to TH and resulted in increased morbidity.

## 6. Splenectomy or Pancreaticosplenectomy in the Treatment of Cancer of the Upper Third of the Stomach

In Japan, pancreaticosplenectomy for LN dissection around the splenic artery (station no. 11) and splenic hilus (station no. 10) had been widely performed, because this procedure was proposed as a radical dissection of metastatic LN along the splenic artery [35, 36]. However, Japanese retrospective analyses proved that there was no survival benefit of these procedures [37, 38]. Recently, pancreas-preserving splenectomy has been considered a safe and effective procedure without decreasing surgical curability [39, 40]. 

In the JCOG 9501 study, pancreas-preserving splenectomy was generally performed with low surgical mortality [27, 41]. In this study, only 22 of 523 patients underwent pancreaticosplenectomy and 59% of patients (13 of 22 cases) developed postoperative complications. 

 In this pancreas-preserving procedure, the splenic artery is generally divided at the distal site after branching-off of the great pancreatic artery in Sasako's modification and the splenic vein is preserved as distal as possible in order to prevent pancreatic fistula and pancreatic atrophy and consequent glucose intolerance [42]. 

In Western countries as well, pancreaticosplenectomy had a marked adverse effect on both mortality and morbidity in two RCTs [5, 6]. 

Currently, pancreaticosplenectomy is considered beneficial only when the primary tumor or metastatic LN directly invades the pancreas, but is not performed for prophylactic dissection of lymph nodes around the splenic artery (station no. 11).

 According to the Japanese experience with LN dissection at the splenic hilus with splenectomy, the incidence of hilar node metastasis ranged 15–21% for tumors located at or infiltrate to the proximal third of the stomach. About 20–25% of patients with LN metastasis have survived over 5 years following LN dissection with splenectomy [35]. However, hilar nodal metastasis was reported to be not found in the early gastric cancer base on retrospective data [43, 44]. Splenectomy is recommended for curative resection of the proximal advanced gastric cancer with infiltration to the greater curvature in the Gastric Cancer Treatment Guidelines 2010 [14].

 Two RCTs compared gastrectomy with splenectomy and gastrectomy alone in patients with gastric cancer were reported with regard to the effectiveness and safety [45, 46].

 Csendes et al. reported 187 patients who underwent total gastrectomy between 1985 and 1992; these patients were randomized into two groups, gastrectomy with splenectomy and gastrectomy alone. Postoperative complications were more frequent in the splenectomy group than in the surgery alone group, including postoperative fever over 38°C (50% versus 39%: *P* < .04), pulmonary complications (39% versus 24%: *P* < .008), and subphrenic abscess (11% versus 4%: *P* < .05). There were no significant differences between the groups in hospital mortality (4.4% for splenectomy versus 3.1% for gastrectomy alone) or in the 5-year survival rate (42% for splenectomy versus 36% for gastrectomy alone) [45].

 The other trial reported by Yu et al. was carried out in Korea between 1995 and 1999. Two hundred seven patients with gastric cancer were divided randomly into two groups, total gastrectomy (103 patients) and total gastrectomy plus splenectomy (104 patients). Postoperative mortality was 8.7% in total gastrectomy alone group and 15.4% in total gastrectomy plus splenectomy group, but there was no significant difference between the groups. Hospital mortality was 1.0% in total gastrectomy alone and 1.9% in total gastrectomy plus splenectomy group; there was no significant difference between the two groups.

 The 5-year survival rates did not differ statistically between the gastrectomy alone group (48.8%) and gastrectomy plus splenectomy group (54.8%). There was no 5-year survivor among patients with lymph node metastasis at the splenic hilum in either group [46]. 

 Therefore, these results did not support the effectiveness of prophylactic dissection at the splenic hilum during splenectomy in patients undergoing total gastrectomy for proximal gastric cancer.

## 7. Future Perspectives

 In Japan and Korea, gastrectomy with D2 LN dissection is the gold standard of treatment for advanced gastric cancer. In order to improve the prognosis of these patients, adjuvant chemotherapy after D2 gastrectomy is thought to be effective and several studies have been reported [47, 48]. Recently, a meta-analysis based on the individual data of 3838 patients from 17 different trials with median follow-up 7 years was reported and indicated a modest but statistically significant benefit associated with adjuvant chemotherapy after curative resection of gastric cancer [49]. In Japan, adjuvant chemotherapy with S-1 is a standard treatment for patients with stage II/III gastric cancer after curative gastrectomy with D2 LN dissection [48]. Moreover, to improve the survival of patients with advanced gastric cancer, neoadjuvant chemotherapy and/or chemotherapy with combination setting or new agents, such as molecular targeting agents, are thought to be necessary in addition to performing D2 gastrectomy with safety and reliability [50].

 Last year, the Japanese Classification of Gastric Carcinoma was revised to conform with the TNM classification of UICC in many respects. In the new guidelines for the Diagnosis and Treatment of Carcinoma of the Stomach, D1, D1+, and D2 gastrectomy were described according to the type of gastrectomy, making the guidelines easier to understand. A global study using unified criteria is necessary to establish a safe and effective worldwide treatment standard including gastrectomy with LN dissection.

## Figures and Tables

**Table 1:** Regional lymph nodes.

No. 1	Right paracardial LN
No. 2	Lest paracardial LN
No. 3a	LN along the left gastric vessels
No. 3b	LN along the right gastric vessels
No. 4sa	LN along the short gastric vessels
No. 4sb	LN along the left gastroepiploic vessels
No. 4d	LN along the right gastroepiploic vessels
No. 5	Suprapyloric LN
No. 6	Infrapyloric LN
No. 7	LN along the left gastric artery
No. 8a	LN along the common hepatic artery (anterosuperior group)
No. 8b	LN along the common hepatic artery (posterior group)
No. 9	LN along the celiac artery
No. 10	LN at the splenic hilum
No. 11p	LN along the proximal splenic artery
No. 11d	LN along the distal splenic artery
No. 12a	LN in the hepatoduodenal ligament (along the hepatic artery)
No. 12b	LN in the hepatoduodenal ligament (along the bile duct)
No. 12p	LN in the hepatoduodenal ligament (behind the portal vain)
No. 13	LN on the posterior surface of the pancreatic head
No. 14v	LN along the superior mesenteric vein
No. 14a	LN along the superior mesenteric artery
No. 15	LN along the middle colic vessels
No. 16a1	LN in the aortic hiatus
No. 16a2	LN around the abdominal aorta (from the upper margin of the celiac trunk to the lower margin of the left renal vein)
No. 16b1	LN around the abdominal aorta (from the lower margin of the left renal vein to the upper margin of the inferior mesenteric artery)
No. 16b2	LN around the abdominal aorta (from the upper margin of the inferior mesenteric artery to the aortic bifurcation)
No. 17	LN on the anterior surface of the pancreas head
No. 18	LN along the inferior margin on the pancreas
No. 19	Infradiaphragmatic LN
No. 20	LN in the esophageal hiatus of the diaphragm
No. 110	Paraesophageal LN in the lower thorax
No. 111	Supradiaphragmatic LN
No. 112	Posterior mediastinal LN

**Table 2:** Depth of tumor invasion (T)—Japanese classification and TNN.

Depth of tumor invasion (T)	Japanese classification (JC: 13th edition)	TNM classification (6th edition)	JC (14th edition)/TNM (7th edition)
Mucosa and/or muscularis mucosa (M)	T1 (M)	Tis/T1	Tis/T1a
Submucosa (SM)	T1 (SM)	T1	T1b
Muscularis propria (MP)	T2 (MP)	T2a	T2
Subserosa (SS)	T2 (SS)	T2b	T3
Penetration of serosa (SE)	T3	T3	T4a
Invasion of adjacent structures (SI)	T4	T4	T4b

**Table 3:** Extent of lymph node metastasis (*N*)—Japanese classification and TNN classification.

*N* category	Japanese classification (JC: 13th edition)	TNM classification (6th edition)	JC (14th edition)/TNM (7th edition)
N_0_	No evidence of LN metastasis	No evidence of LN metastasis	No evidence of LN metastasis
N_1_	Metastasis to only Group 1 LN	Metastasis in 1 to 6 regional LNs	Metastasis in 1 to 2 regional LNs
N_2_	Metastasis to Group 2 LN, but no metastasis to Group 3 LN	7–15 nodes	3–6 nodes
N_3_	Metastasis to Group 3 LN	16 or more nodes	7 or more nodes N3a: 7–15 nodes N3b: 16 or more nodes

LN: lymph node.

**Table 4:** Randomized controlled trials comparing D1 with D2/D3.

Study	Country	Comparison	Postoperative morbidity	Postoperative mortality	5-year survival
Dutch trial (1989–1993)	Netherlands	D1 (*n* = 380) D2 (*n* = 331)	25% 43% (*P* < .001)	4% 10% (*P* = .004)	45% 47% HR 1.00 (95% CI, 0.82–1.22)
MRC trial (1987–1994)	UK	D1 (*n* = 200) D2 (*n* = 200)	28% 46% (*P* < .001)	6.5% 13% (*P* = .04)	35% 33% HR 1.10 (95% CI, 0.87–1.39)
Taiwanese trial (1993–1999)	Taiwan	D1 (*n* = 110) D3 (*n* = 111)	7.3% 17.1% (*P* = .012)	0% 0%	53.6% 59.5% HR 0.49 (95% CI, 0.32–0.77)
IGCSG trial (1999–2002)	Italy	D1 (*n* = 76) D2 (*n* = 86)	10.5% 16.3% (*P* < .029)	0% 1.3% (N.S)	Under analysis

**Table 5:** Randomized controlled trials comparing D2 with D2 plus para-aortic lymph nodes.

Study	Country	Comparison	Postoperative morbidity	Postoperative mortality	5-year survival
JCOG trial (1995–2001)	Japan	D2 (*n* = 263) D2+ PALN (*n* = 260)	20.9% 28.1% (*P* = .067)	0.8% 0.8% (*P* = .99)	69.2% 70.3% HR 1.03 (95% CI, 0.77–1.37)
Polish trial (1999–2003)	Poland	D2 (*n* = 141) D2+ PALN (*n* = 134)	27.7% 21.6% (*P* = .248)	4.9% 2.2% (*P* = .37)	Under analysis
East Asian trial (1995–2002)	Japan, Korea, and Chinese Taiwan area	D2 (*n* = 135) D2+ PALN (*n* = 134)	26% 39% (*P* = .023)	0.7% 3.7% (*P* = .107)	52.6% 55.4% (*P* = .801)

D2: gastrectomy with D2 lymph node dissection. PALN: para-aortic lymph node dissection.
